# Supplementary material for: The long noncoding RNA lncNB1 promotes tumorigenesis by interacting with ribosomal protein RPL35
Source: Nat Commun. 2019 Nov 5;10:5026. doi: 10.1038/s41467-019-12971-3 (PMC6831662; doi:10.1038/s41467-019-12971-3)
Supplement: Supplementary file 1 — Supplementary Information [file 41467_2019_12971_MOESM1_ESM.pdf]

Supplementary Information

The long noncoding RNA lncNB1 promotes tumorigenesis by interacting with ribosomal protein RPL35

Liu et al.

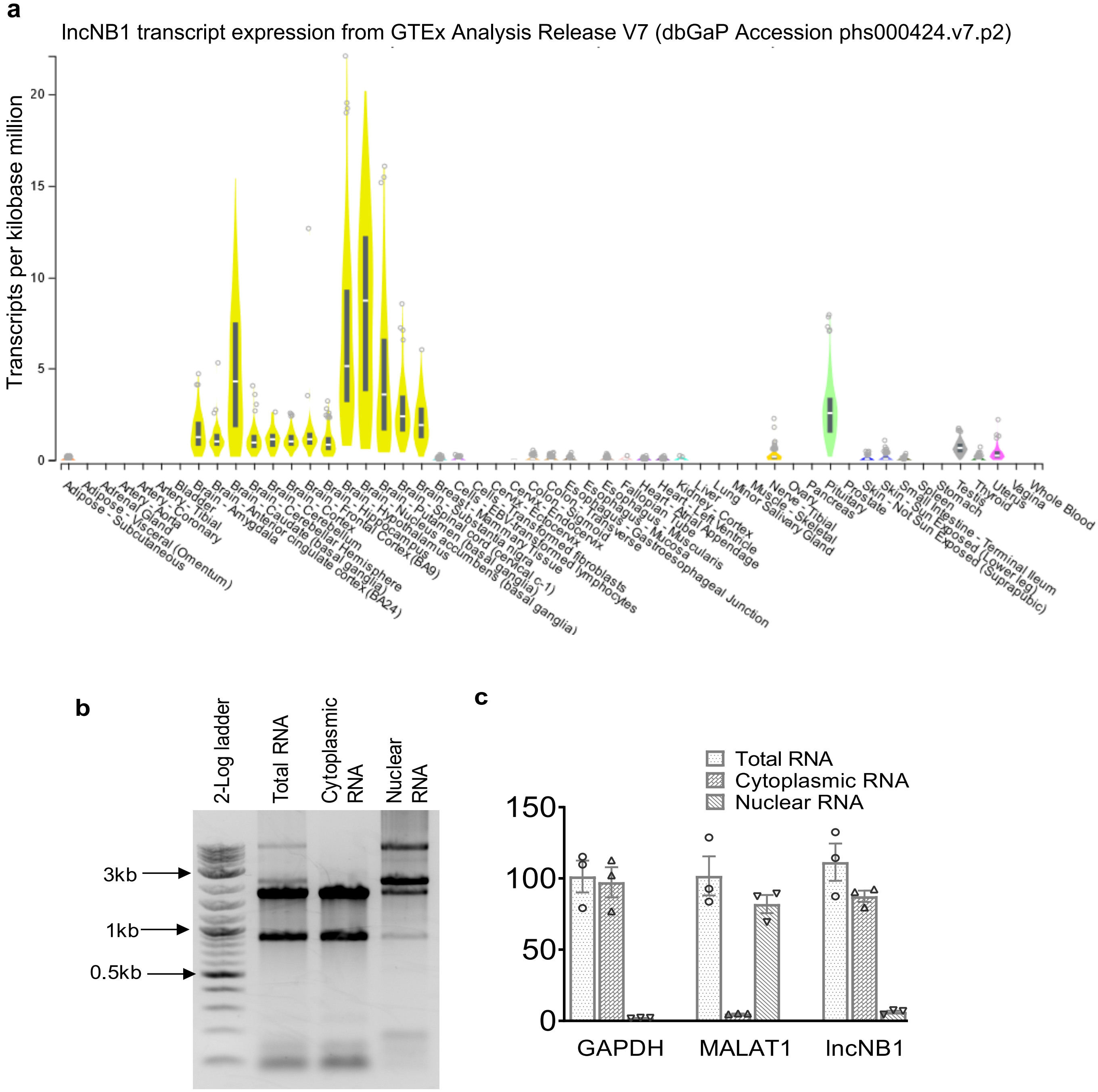

Supplementary Fig. 1. lncNB1 is expressed in normal brain, pituitary, testis, uterus and nerve tissues and is mainly localized in the cytoplasm. **a**, lncNB1 transcript expression levels in normal tissues were directly obtained from the publicly available Genotype-Tissue Expression (GTEx) Release V7 dataset. Box plots were shown as median and 25th and 75th percentiles. Points were displayed as outliers if they were above or below 1.5 times the interquartile range. **b-c**, RNA was extracted from BE(2)-C cells with or without cytoplasmic and nuclear RNA fractionation. The RNA samples were run on an acrylamide gel (**b**) or subjected to RT-PCR analysis of lncNB1, the cytoplasmic RNA marker GAPDH and the nuclear RNA marker MALAT1 (**c**). Data were shown as the mean ± standard error of three independent experiments. Error bars represented standard errors. Source data are provided as a Source Data file.

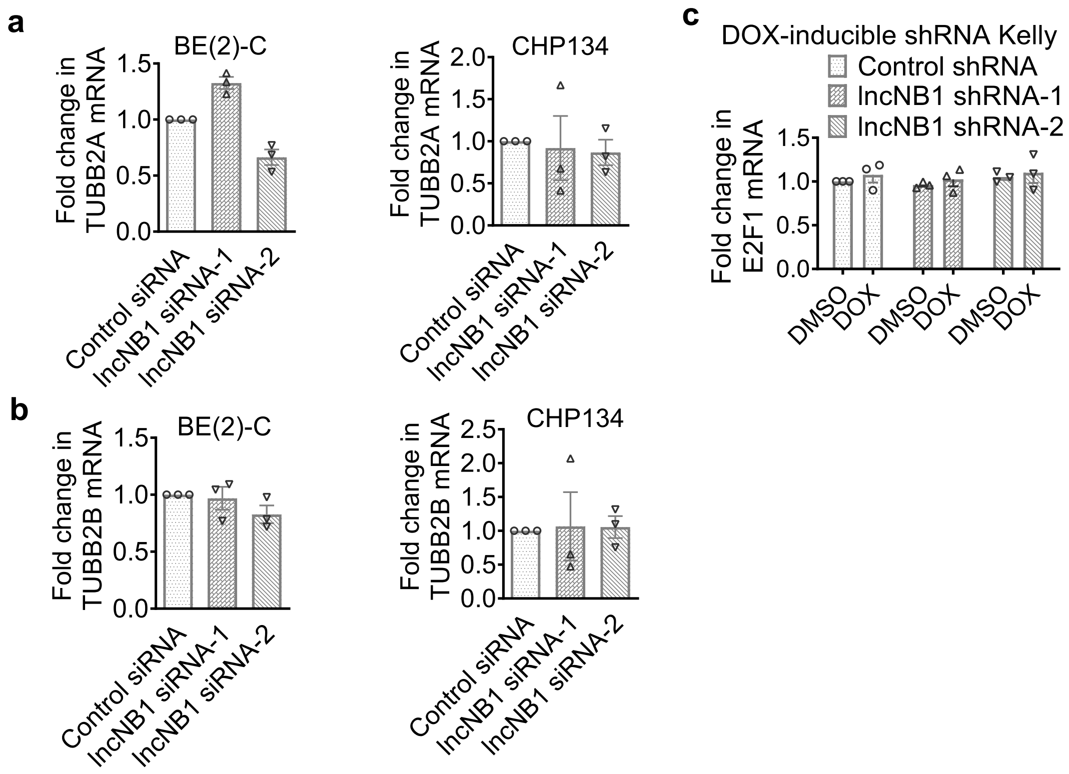

Supplementary Fig. 2. lncNB1 does not regulate the expression of its neighboring protein coding genes TUBB2A and TUBB2B and does not regulate E2F1 mRNA expression. **a-b**, BE(2)-C and CHP134 cells were transfected with control siRNA, lncNB1 siRNA-1 or lncNB1 siRNA-2 for 48 hours, followed by RNA extraction and RT-PCR analysis of TUBB2A (**a**) and TUBB2B (**b**) mRNA expression. Data were shown as the mean ± standard error of three independent experiments, and evaluated by one-way ANOVA. **c**, Doxycycline (DOX)-inducible control shRNA, lncNB1 shRNA-1 or lncNB1 shRNA-2 Kelly cells were treated with vehicle control or DOX for 48 hours, followed by RT-PCR analysis of E2F1 mRNA expression. Data were shown as the mean ± standard error of three independent experiments. Error bars represented standard errors. Source data are provided as a Source Data file.

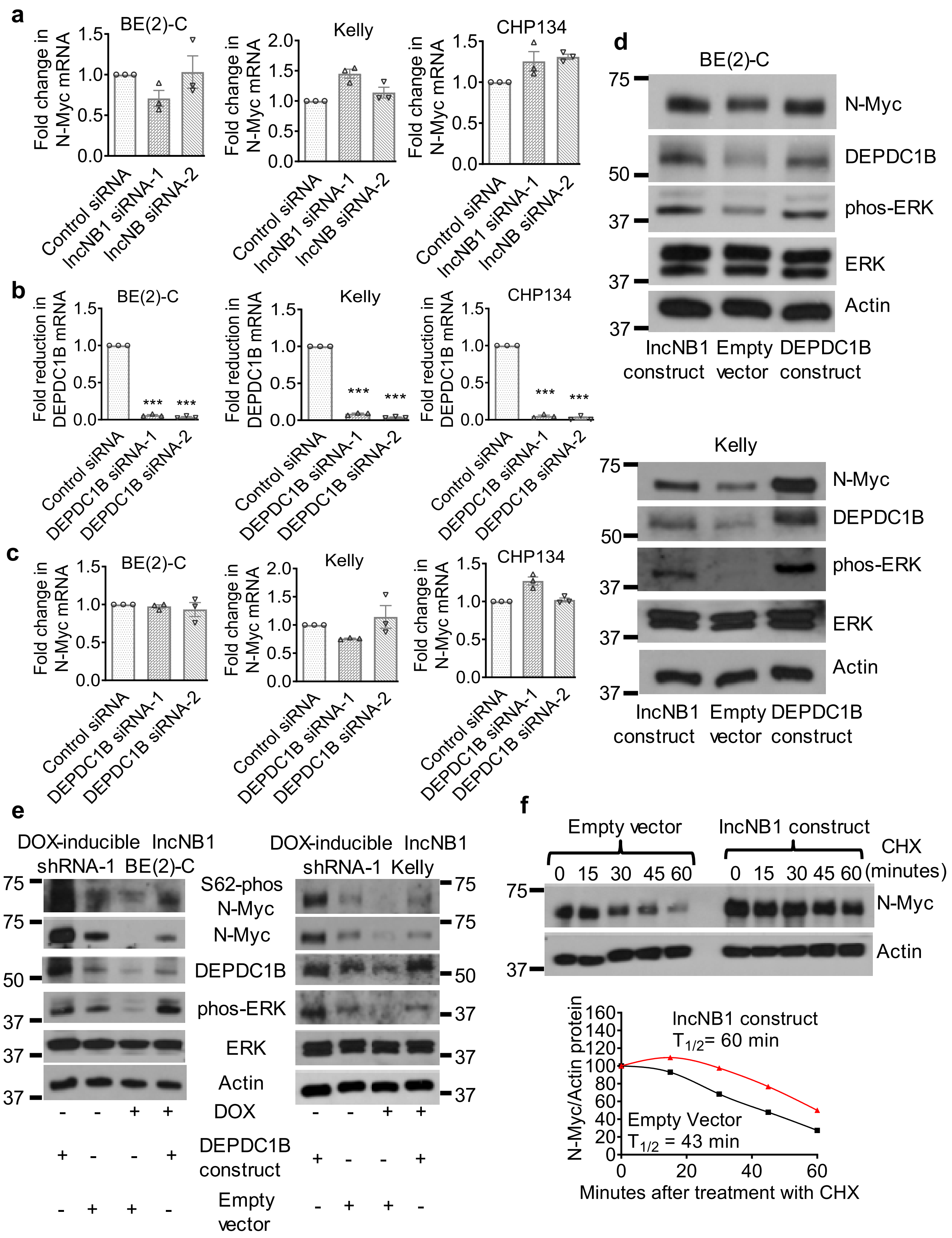
a
BE(2)-C
Kelly
CHP134
Fold change in N-Myc mRNA
Control siRNA
lncNB1 siRNA-1
lncNB siRNA-2
b
Fold reduction in DEPDC1B mRNA
DEPDC1B siRNA-1
DEPDC1B siRNA-2
***
c
d
N-Myc
DEPDC1B
phos-ERK
ERK
Actin
lncNB1 construct
Empty vector
DEPDC1B construct
e
DOX-inducible lncNB1 shRNA-1 BE(2)-C
DOX-inducible lncNB1 shRNA-1 Kelly
S62-phos N-Myc
DOX
DEPDC1B construct
Empty vector
f
Empty vector
lncNB1 construct
CHX (minutes)
0 15 30 45 60
N-Myc/Actin protein
lncNB1 construct
T1/2= 60 min
Empty Vector
T1/2 = 43 min
Minutes after treatment with CHX

Supplementary Fig. 3. LncNB1 and DEPDC1B up-regulate N-Myc protein but not mRNA expression. **a,** BE(2)-C, Kelly and CHP134 cells were transfected with control siRNA, lncNB1 siRNA-1 or lncNB1 siRNA-2 for 48 hours, followed by RT-PCR analysis of N-Myc mRNA expression. **b-c**, BE(2)-C, Kelly and CHP134 cells were transfected with control siRNA, DEPDC1B siRNA-1 or DEPDC1B siRNA-2 for 48 hours, followed by RT-PCR analysis of DEPDC1B (**b**) and N-Myc (**c**) mRNA expression. Data were shown as the mean ± standard error of three independent experiments, and evaluated by one-way ANOVA. *** $P < 0.001$. **d,** BE(2)-C and Kelly cells were transfected with an empty vector, lncNB1 or DEPDC1B expression construct for 48 hours, followed by immunoblot analysis of DEPDC1B, phosphorylated (phos)-ERK, ERK and N-Myc proteins. **e**, DOX-inducible lncNB1 shRNA-1 BE(2)-C and Kelly cells were transfected with an empty vector or DEPDC1B expression construct and treated with vehicle control or DOX for 48 hours, followed by immunoblot analysis of DEPDC1B, ERK, phos-ERK, S62-phos-N-Myc and total N-Myc proteins. **f,** BE(2)-C cells were transfected with an empty vector or lncNB1 expression construct for 48 hours, and treated with 50µM cycloheximide (CHX) for the last 0, 15, 30, 45 or 60 minutes. Protein was extracted for immunoblot analysis of N-Myc. N-Myc protein level was normalized by actin, and N-Myc protein half-life ($T_{1/2}$) was obtained from the line chart. Source data are provided as a Source Data file.

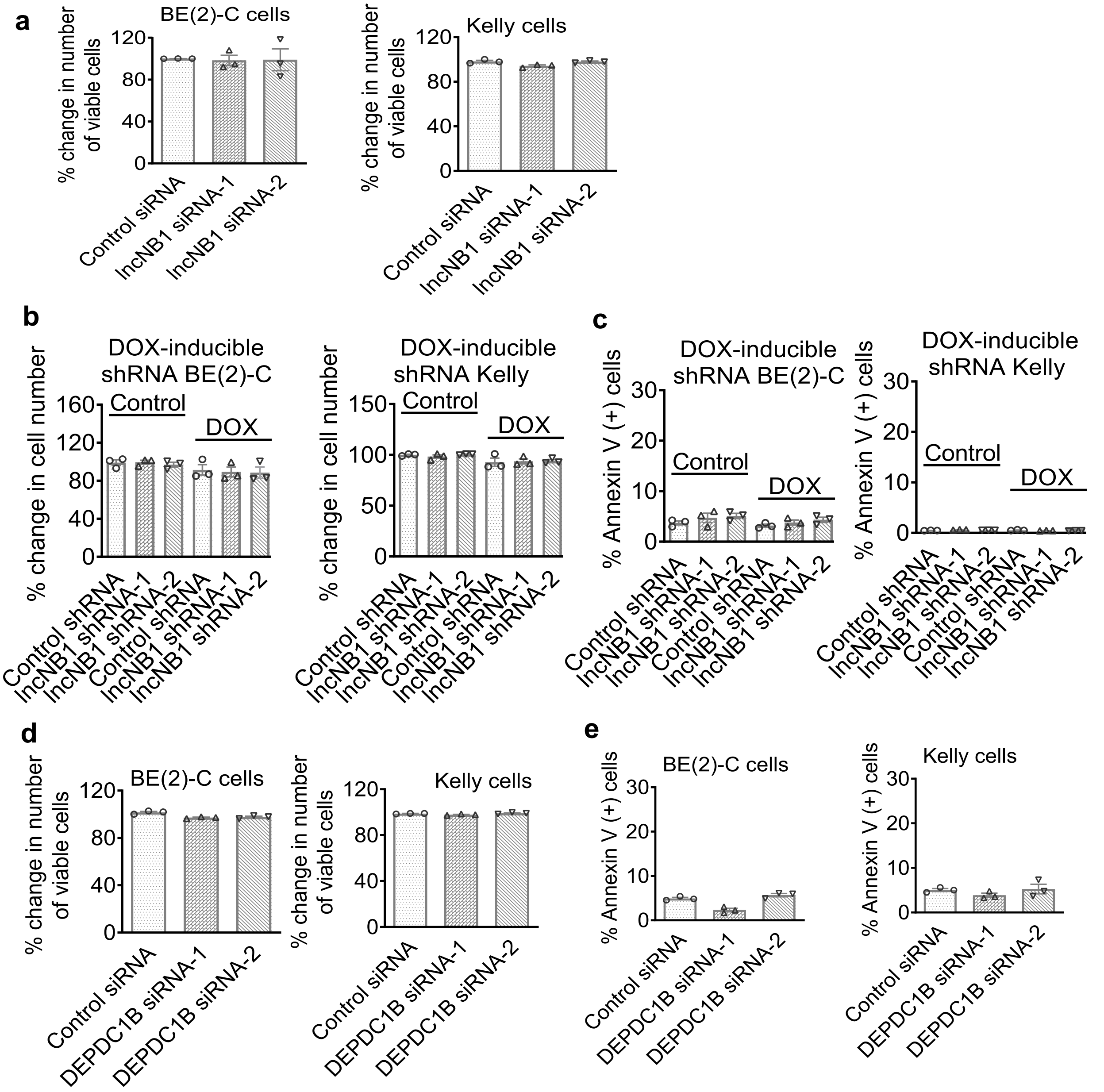

Supplementary Fig. 4. Knocking down lncNB1 or DEPDC1B for 48 hours is too early to have an effect on neuroblastoma cell proliferation or survival. **a,** BE(2)-C and Kelly cells were transfected with control siRNA, lncNB1 siRNA-1 or lncNB1 siRNA-2 for 48 hours, followed by Alamar blue assays of relative numbers of cells. **b-c**, DOX-inducible control shRNA, lncNB1 shRNA-1 or lncNB1 shRNA-2 BE(2)-C and Kelly cells were treated with vehicle control or DOX for 48 hours, followed by Alamar blue assays of relative numbers of cells (**b**) or staining with Annexin V and flow cytometry analysis of Annexin V-positive apoptotic cells (**c**). **d-e,** BE(2)-C and Kelly cells were transfected with control siRNA, DEPDC1B siRNA-1 or DEPDC1B siRNA-2 for 48 hours, followed by Alamar blue assays of relative numbers of cells (**d**) or staining with Annexin V and flow cytometry analysis of Annexin V-positive apoptotic cells (**e**). Throughout, data were shown as the mean ± standard error of three independent experiments, and evaluated by two-sided unpaired Student's *t*-test for two groups or one-way ANOVA for more than two groups. Source data are provided as a Source Data file.

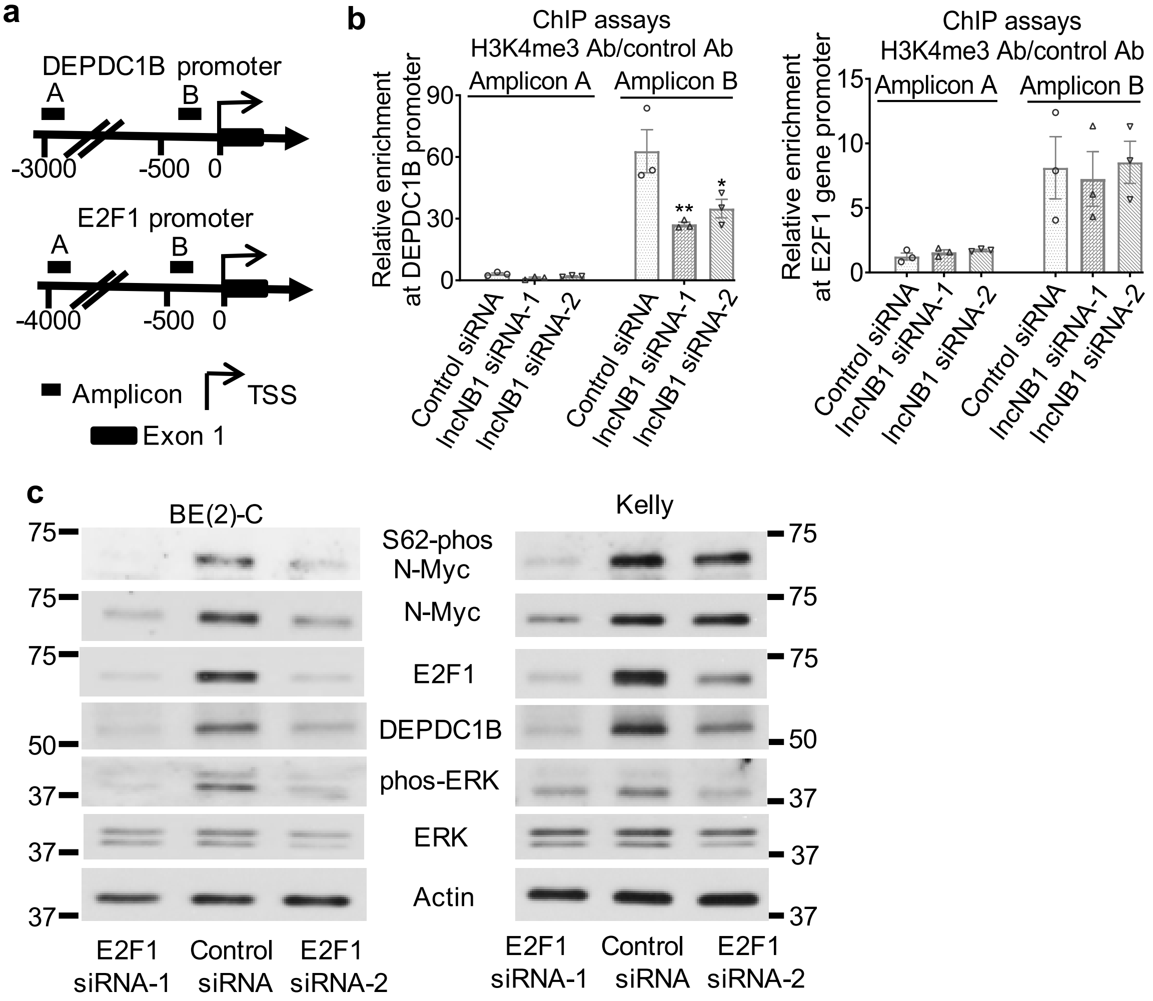

Supplementary Fig. 5. LncNB1 induces *DEPDC1B* gene transcription through E2F1. **a**, Schematic representation of the *DEPDC1B* and *E2F1* gene promoter regions. TSS represented transcription start sites. Amplicons indicated the positions for PCR primers. **b**, ChIP assays were performed with a control antibody or an anti-trimethyl-H3K4 (H3K4me3) antibody and real-time PCR with primers targeting the far upstream or proximal *DEPDC1B* or *E2F1* gene promoter in BE(2)-C cells after transfection with control siRNA, lncNB1 siRNA-1 or lncNB1 siRNA-2 for 48 hours. Fold change in H3K4me3 occupancy at the *DEPDC1B* or *E2F1* gene promoter was obtained, after dividing PCR products from samples immunoprecipitated with the H3K4me3 antibody by those with the control antibody and normalized to a negative control region. Data were shown as the mean ± standard error of three independent experiments, and evaluated by one-way ANOVA. * and ** indicates $P < 0.05$ and 0.01 respectively. **c**, BE(2)-C and Kelly cells were transfected with control siRNA, E2F1 siRNA-1 or E2F1 siRNA-2 for 48 hours, followed by immunoblot analysis of E2F1, DEPDC1B, ERK, phosphorylated (phos)-ERK, S62-phos-N-Myc and total N-Myc proteins. Source data are provided as a Source Data file.

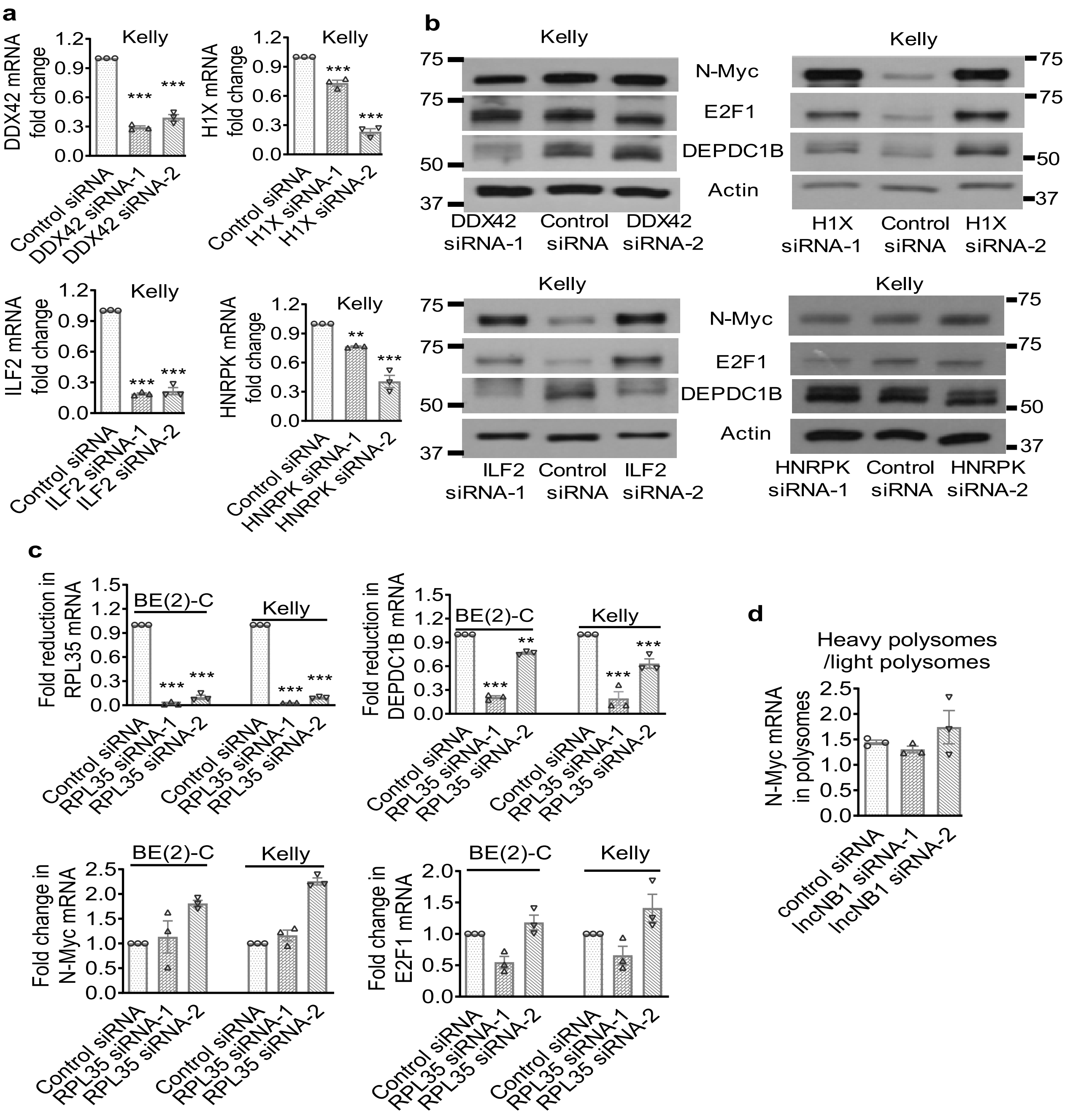
a
Kelly
DDX42 mRNA fold change
Control siRNA
DDX42 siRNA-1
DDX42 siRNA-2
H1X mRNA fold change
H1X siRNA-1
H1X siRNA-2
ILF2 mRNA fold change
ILF2 siRNA-1
ILF2 siRNA-2
HNRPK mRNA fold change
HNRPK siRNA-1
HNRPK siRNA-2
b
N-Myc
E2F1
DEPDC1B
Actin
75
50
37
DDX42 siRNA-1
Control siRNA
DDX42 siRNA-2
H1X siRNA-1
H1X siRNA-2
ILF2 siRNA-1
ILF2 siRNA-2
HNRPK siRNA-1
HNRPK siRNA-2
c
BE(2)-C
Fold reduction in RPL35 mRNA
Fold reduction in DEPDC1B mRNA
Fold change in N-Myc mRNA
Fold change in E2F1 mRNA
RPL35 siRNA-1
RPL35 siRNA-2
d
Heavy polysomes /light polysomes
N-Myc mRNA in polysomes
control siRNA
lncNB1 siRNA-1
lncNB1 siRNA-2

Supplementary Fig. 6. Knocking down DDX42, H1X, ILF2 and HNRPK does not consistently reduce DEPDC1B, N-Myc and E2F1 protein expression, and knocking down RPL35 reduces DEPDC1B but not N-Myc and E2F1 mRNA expression. **a-b**, Kelly cells were transfected with control siRNA, DDX42 siRNA-1 or siRNA-2, H1X siRNA-1 or siRNA-2, ILF2 siRNA-1 or siRNA-2, or HNRPK siRNA-1 or siRNA-2 for 48 hours, followed by RNA extraction and RT-PCR analysis of DDX42, H1X, ILF2 or HNRPK mRNA expression (**a**), or followed by protein extraction and immunoblot analysis of DEPDC1B, N-Myc and E2F1 protein expression (**b**). **c**, BE2C and Kelly cells were transfected with control siRNA, RPL35 siRNA-1 or RPL35 siRNA-2 for 48 hours, followed by RT-PCR analysis of RPL35, DEPDC1B, N-Myc and E2F1 mRNA expression. **d**, BE(2)-C cells were transfected with control siRNA, lncNB1 siRNA-1 or lncNB1 siRNA-2 for 48 hours, followed by treatment with 50 μg/ml of cycloheximide and polysome fractionation. RT-PCR analysis of N-Myc mRNA was performed and results were pooled into light (fraction 7, 8 and 9) and heavy (fraction 12, 13 and 14) polysomes and expressed as the ratio of mRNA in heavy polysome and mRNA in light polysome. Data were shown as the mean ± standard error of three independent experiments and evaluated by one-way ANOVA. ** and *** indicate $P < 0.01$ and 0.001 respectively. Source data are provided as a Source Data file.

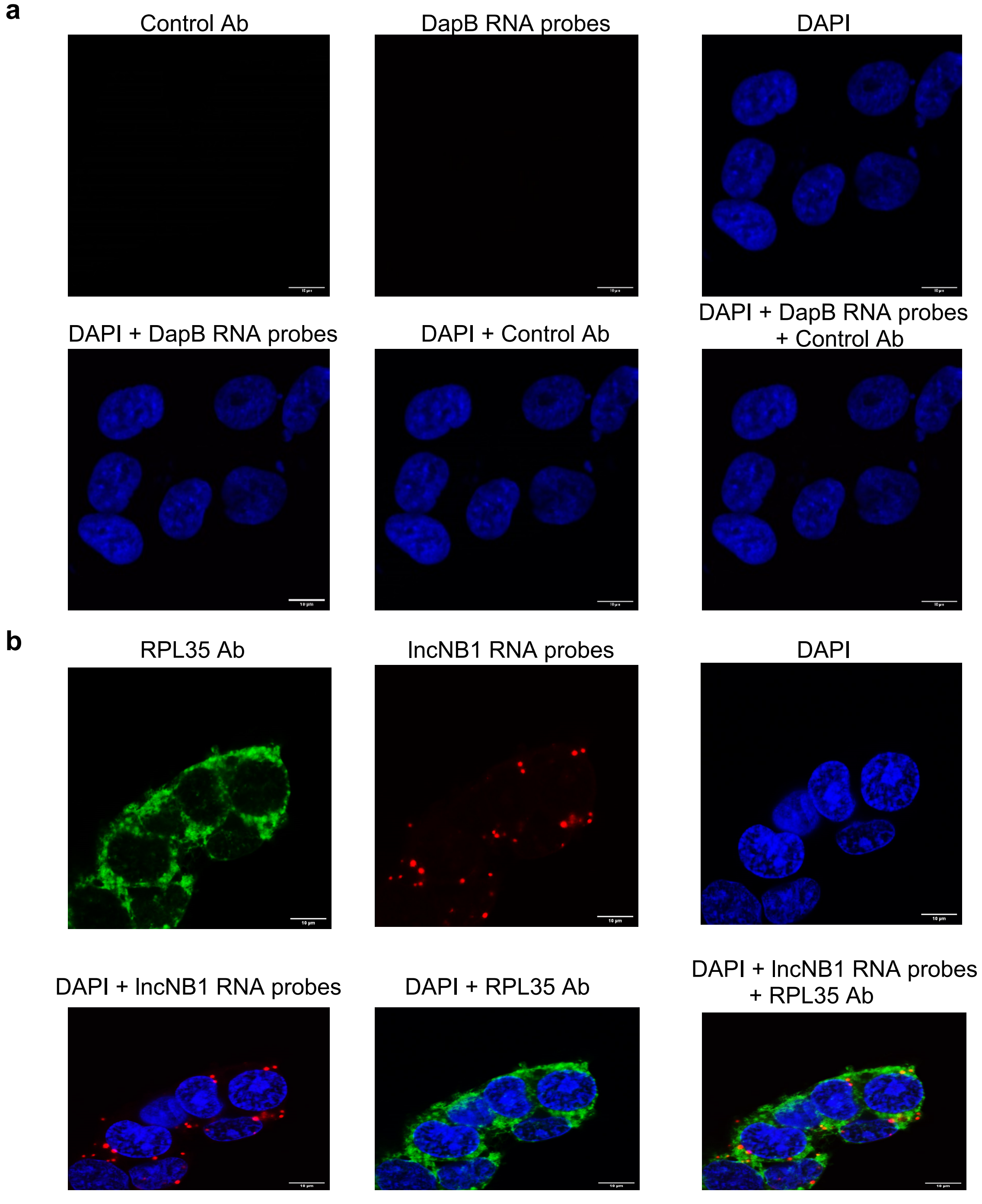

Supplementary Fig. 7. lncNB1 RNA and RPL35 protein colocalize in the cytoplasm. **a-b**, BE(2)-C cells were seeded into 8 well chamber slides for 48 hours, followed by fluorescent in situ hybridization assays with the negative control RNA probe targeting DapB RNA (**a**) or lncNB1 RNA probe targeting the 108-1169 region of lncNB1 RNA (**b**). The cells were then immunostained with rabbit control antibody (Ab) (**a**) or rabbit anti-RPL35 Ab (**b**). Nuclei were counter stained with DAPI. Scale bar = 10 μM.

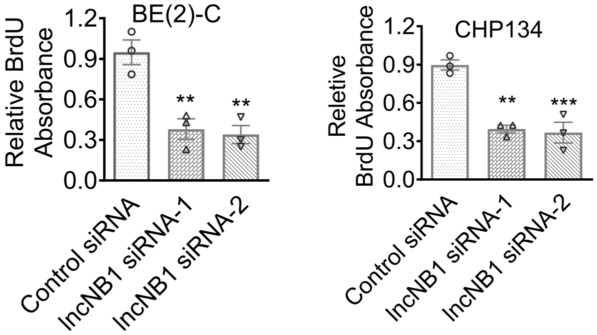

Supplementary Fig. 8. Knocking down lncNB1 reduces neuroblastoma cell proliferation. BE(2)-C and CHP134 cells were transfected with control siRNA, lncNB1 siRNA-1 or lncNB1 siRNA-2 for 96 hours, followed by incubation with BrdU in the last five hours and BrdU incorporation assays. Data were shown as the mean ± standard error of three independent experiments, and evaluated by one-way ANOVA. ** and *** indicates $P < 0.01$ and 0.001 respectively. Source data are provided as a Source Data file.

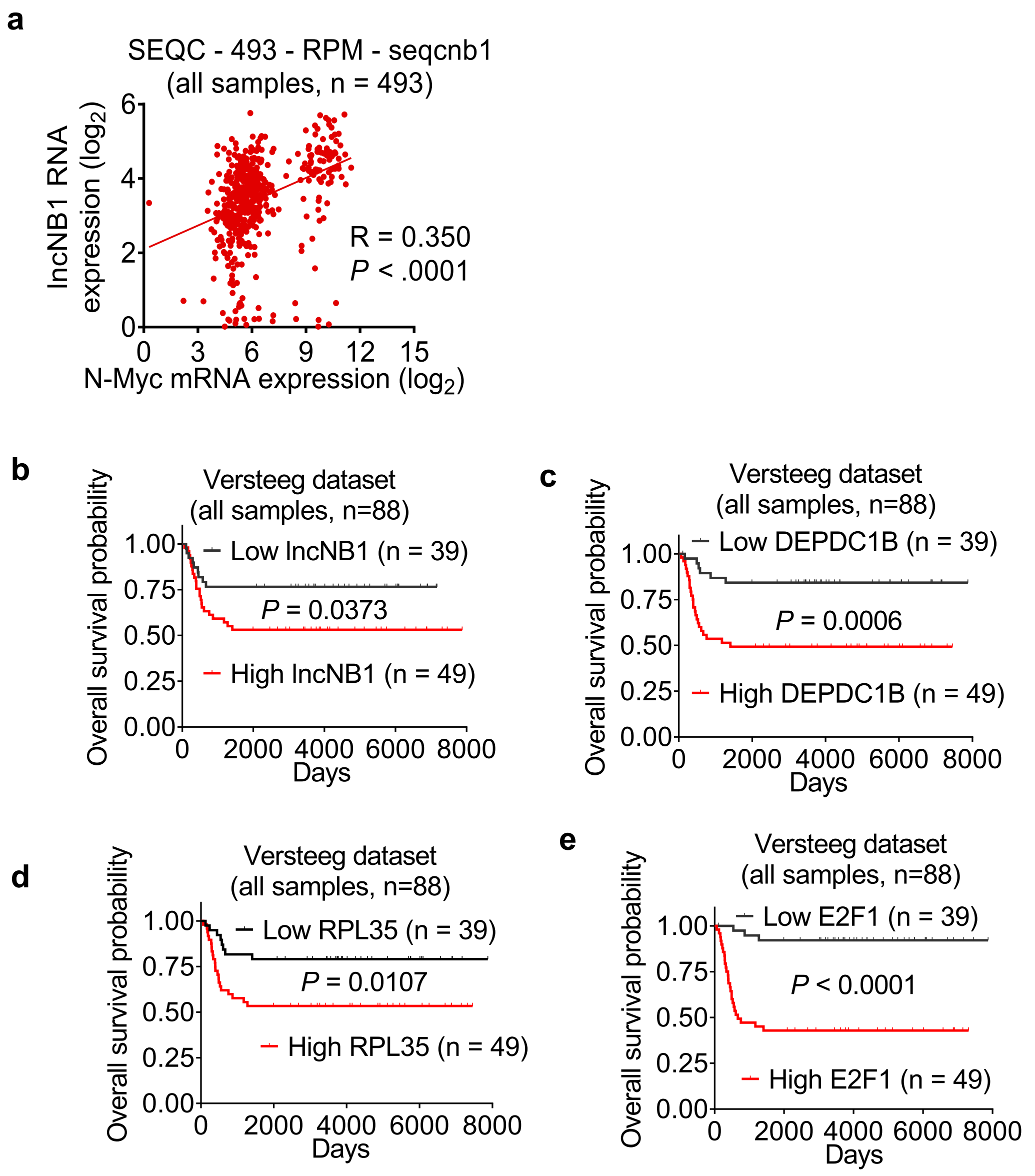

Supplementary Fig. 9. High levels of lncNB1, DEPDC1B, E2F1 and RPL35 expression neuroblastoma tissues predict poor patient prognosis. **a**, Two-sided Pearson's correlation was employed to analyze the association between lncNB1 RNA expression and N-Myc mRNA expression in 493 human neuroblastoma tissues in the publicly available RNA sequencing gene expression SEQC-RPM-seqcnb1 downloaded from the R2 platform (http://r2.amc.nl). **b-e**, Kaplan–Meier curves showed the probability of overall survival of neuroblastoma patients according to the levels of lncNB1 (**b**), DEPDC1B (**c**), RPL35 (**d**) and E2F1 (**e**) expression in the 88 tumor tissues of the Versteeg dataset using the two-sided log-rank test. Source data are provided as a Source Data file.

Supplementary Table 1. LncNB1 gene copy number variation in human neuroblastoma tissues from 7 out of 341 patients, as analysed by SNP array data originally generated by the Therapeutically Applicable Research to Generate Effective Treatments (TARGET) initiative (https://target-data.nci.nih.gov/). LncNB1 was named LOC100507194 by the TARGET dataset.

**Samples**	**Chromosome**	**Position**	**Numer of SNPs**	**RegionLength**
./CNV/6258_0478-T-D.txt	chr6	110391-19336917	3817	19,226,527
./CNV/6258_1599-T-D.txt	chr6	1717719-4336871	641	2,619,153
./CNV/6258_3753-T-D.txt	chr6	2392698-5309289	650	2,916,592
./CNV/6258_0292-T-D.txt	chr6	3119946-4213757	288	1,093,812
./CNV/6258_1278-T-D.txt	chr6	2986199-3348874	81	362,676
./CNV/6258_2023-T-D.txt	chr6	3132792-3138439	4	5,648
./CNV/6258_1931-T-D.txt	chr6	348799-3261745	802	2,912,947

**Samples**	**Copy Number**	**Start_SNP**	**End_SNP**	**Conf**
./CNV/6258_0478-T-D.txt	3	rs4959515	rs1858513	3040.69
./CNV/6258_1599-T-D.txt	3	rs1040532	rs1023017	409.176
./CNV/6258_3753-T-D.txt	3	rs742487	rs9405828	398.249
./CNV/6258_0292-T-D.txt	3	rs9501935	rs12191799	150.987
./CNV/6258_1278-T-D.txt	3	rs7774283	rs10900941	22.83
./CNV/6258_2023-T-D.txt	1	rs6929360	rs9503437	6.809
./CNV/6258_1931-T-D.txt	3	rs3778607	rs9378784	406.177

**Samples**	**OverLapGenes**
./CNV/6258_0478-T-D.txt	ADTRP,ATXN1,BLOC1S5,BLOC1S5-TXNDC5,BMP6,BPHL,C6orf195,C6orf201,C6orf52,CAGE1,CAP2,CD83,CDYL,DEK,DKFZP686I15217,DSP,DTNBP1,DUSP22,ECI2,EDN1,EEF1E1,EEF1E1-MUTED,ELOVL2,ELOVL2-AS1,ERVFRD-1,EXOC2,F13A1,FAM217A,FAM50B,FAM8A1,FARS2,FOXC1,FOXF2,FOXQ1,GCM2,GCNT2,GFOD1,GMDS,GMPR,HIVEP1,HTATSF1P2,HULC,HUS1B,IRF4,JARID2,KDM1B,KIF13A,LINC00518,LOC100130275,LOC100130357,LOC100506207,lncNB1(LOC100507194),LOC100508120,LOC285768,LY86,LY86-AS1,LYRM4,MAK,MCUR1,MGC39372,MIR3691,MIR4639,MIR4645,MIR548A1,MYLIP,MYLK4,NEDD9,NHLRC1,NOL7,NQO2,NRN1,NUP153,PAK1IP1,PHACTR1,PIP5K1P1,PPP1R3G,PRPF4B,PSMG4,PXDC1,RANBP9,RBM24,RIOK1,RIPK1,RNF144B,RNF182,RPP40,RREB1,SCARNA27,SERPINB1,SERPINB6,SERPINB9,SIRT5,SLC22A23,SLC35B3,SMIM13,SNRNP48,SSR1,STMND1,SYCP2L,TBC1D7,TFAP2A,TMEM14B,TMEM14C,TMEM170B,TPMT,TUBB2A,TUBB2B,TXNDC5,WRNIP1
./CNV/6258_1599-T-D.txt	BPHL,C6orf195,C6orf201,DKFZP686I15217,ECI2,FAM217A,FAM50B,GMDS,HTATSF1P2,lncNB1(LOC100507194),LOC100508120,MGC39372,MIR4645,MYLK4,NQO2,PRPF4B,PSMG4,PXDC1,RIPK1,SERPINB1,SERPINB6,SERPINB9,SLC22A23,TUBB2A,TUBB2B,WRNIP1
./CNV/6258_3753-T-D.txt	BPHL,C6orf195,C6orf201,CDYL,DKFZP686I15217,ECI2,FAM217A,FAM50B,FARS2,HTATSF1P2,lncNB1(LOC100507194),LYRM4,MGC39372,MIR3691,MIR4645,MYLK4,NQO2,PPP1R3G,PRPF4B,PSMG4,PXDC1,RIPK1,RPP40,SERPINB1,SERPINB6,SERPINB9,SLC22A23,TUBB2A,TUBB2B,WRNIP1
./CNV/6258_0292-T-D.txt	C6orf201,ECI2,FAM217A,FAM50B,lncNB1(LOC100507194),PRPF4B,PSMG4,PXDC1,SLC22A23,TUBB2B
./CNV/6258_1278-T-D.txt	BPHL,lncNB1(LOC100507194),PSMG4,RIPK1,SLC22A23,TUBB2A,TUBB2B
./CNV/6258_2023-T-D.txt	lncNB1(LOC100507194)

./CNV/6258_1931-T-D.txt	BPHL,C6orf195,DKFZP686I15217,EXOC2,FOXC1,FOXF2,FOXQ1,GMDS, HTATSF1P2,HUS1B,IRF4,lncNB1(LOC100507194),LOC100508120,LOC285768, MGC39372,MIR4645,MYLK4,NQO2,PSMG4,RIPK1,SERPINB1,SERPINB6, SERPINB9,SLC22A23,TUBB2A,TUBB2B,WRNIP1

Supplementary Table 2. Genes up- or down-regulated by lncNB1 siRNA-1 and lncNB1 siRNA-2 compared with control shRNA, as identified by Affymetrix microarray analysis in BE(2)-C cells after transfection with control siRNA, lncNB1 siRNA-1 or lncNB1 siRNA-2 for 40 hours. The microarray experiments were repeated four times. Differential expression analysis was performed using the Limma package. Moderated t tests were performed with the Limma package. Genes with fold change of > 1.7, p < 0.05 and adjusted p < 0.20 were listed.

Gene Symbol	Probe ID	logFC	Fold Change	P Value	Adjusted P Value
SLC7A11	16979917	1.615424	3.064015	6.19E-12	1.20E-07
DARS2	16673983	-1.986538	-3.96285	1.46E-09	1.42E-05
G3BP1	16991261	-1.50313	-2.83457	1.46E-08	9.47E-05
PI15	17070110	1.011078	2.015416	3.28E-08	0.0001589
TRPM7	16809138	-1.537074	-2.90205	2.25E-07	0.0008715
USP13	16948320	-1.498519	-2.82553	7.70E-07	0.0024129
KIF20A	16989636	-1.325458	-2.50612	1.37E-06	0.0024129
PLK4	16970563	-1.321082	-2.49853	1.16E-06	0.0024129
TRAPPC6B	16792268	0.949246	1.930864	1.04E-06	0.0024129
SMIM15	16996605	0.957132	1.941446	1.32E-06	0.0024129
MIGA1	16666392	0.990396	1.98673	9.88E-07	0.0024129
ARL6IP6	16886602	-1.399314	-2.63776	1.80E-06	0.0026557
HIST1H3B	17016363	-1.101536	-2.14583	2.05E-06	0.0026557
ANO3	16723020	0.886242	1.848355	1.97E-06	0.0026557
HTN3	16967398	0.964393	1.951242	1.96E-06	0.0026557
GABRG2	16991816	0.839633	1.789595	2.56E-06	0.0029244
GPR85	17061992	0.974359	1.964768	2.49E-06	0.0029244
SLC35A5	16943954	-1.31718	-2.49179	3.17E-06	0.0031487
RBPMS2	16810572	-1.224194	-2.33625	3.02E-06	0.0031487
CENPQ	17009482	-1.186976	-2.27675	3.25E-06	0.0031487
IRF6	16698923	1.021947	2.030658	4.06E-06	0.0037497
TARDBP	16658926	-1.278089	-2.42518	4.68E-06	0.0037806
HIPK1	16668969	-1.172578	-2.25414	4.67E-06	0.0037806
E2F8	16736638	-1.12551	-2.18179	4.66E-06	0.0037806
NCAPD2	16747287	-1.20363	-2.30319	4.99E-06	0.0038704
ESCO2	17067332	-1.257754	-2.39123	5.54E-06	0.0041337
SLITRK6	16780133	0.86138	1.816775	7.02E-06	0.0048647
ABCA12	16907979	0.94142	1.920418	6.88E-06	0.0048647
TXNDC16	16792998	-1.117576	-2.16982	7.93E-06	0.0053035
EIF1AX	17109706	-1.327706	-2.51003	8.76E-06	0.0054828
EDC3	16811672	-1.133156	-2.19338	8.72E-06	0.0054828
CAB39L	16779059	-1.166832	-2.24518	9.70E-06	0.0055042
FAM171B	16888554	-1.082339	-2.11747	9.68E-06	0.0055042
DENND6A	16955456	-0.950091	-1.93199	9.20E-06	0.0055042
TMTC1	16762759	0.836206	1.785349	9.93E-06	0.0055042
DZIP1	16780322	-1.086648	-2.1238	1.10E-05	0.0059503
CENPI	17105401	-1.082589	-2.11783	1.31E-05	0.0068684
KIF18A	16736891	-1.309192	-2.47803	1.63E-05	0.0083414
XRCC2	17064679	-1.141425	-2.20599	1.82E-05	0.0088172
SYNPO2	16970118	-0.958447	-1.94322	1.80E-05	0.0088172
MIS18BP1	16792381	-1.141465	-2.20605	1.99E-05	0.0089657

REEP3	16705260	-1.052808	-2.07456	1.98E-05	0.0089657
GABRA4	16975642	-0.959989	-1.9453	1.93E-05	0.0089657
GPT2	16818610	0.822163	1.768054	2.27E-05	0.0100116
ATP10A	16806145	0.801853	1.743339	2.41E-05	0.01038
STIL	16686796	-1.249001	-2.37677	2.52E-05	0.0104958
KIF20B	16707221	-1.141688	-2.20639	2.54E-05	0.0104958
KIF2C	16663958	-1.134186	-2.19495	2.74E-05	0.0110569
ULBP1	17013657	0.799979	1.741076	2.90E-05	0.0114732
CKAP2	16775014	-1.118913	-2.17183	3.10E-05	0.0120388
PRKAR2B	17050154	-1.109705	-2.15801	3.57E-05	0.0135647
INTS14	16810828	-1.053866	-2.07609	4.01E-05	0.0149452
ASAP1	17081162	-1.22981	-2.34536	4.22E-05	0.0149461
SOSTDC1	17055472	-1.075417	-2.10733	4.24E-05	0.0149461
FAM96A	16810503	-1.033863	-2.0475	4.15E-05	0.0149461
DPY19L4	17070972	-1.08561	-2.12227	4.57E-05	0.0151774
MAGED1	17103804	-0.964333	-1.95116	4.62E-05	0.0151774
SFT2D2	16673477	-0.951999	-1.93455	4.54E-05	0.0151774
PDZD11	17111808	-0.875902	-1.83516	4.54E-05	0.0151774
PUS7	17061467	-1.23273	-2.35011	5.04E-05	0.0163003
TUBD1	16847267	-1.070372	-2.09997	5.12E-05	0.0163003
FAM167A	17074641	-0.971093	-1.96033	5.29E-05	0.016567
UTP18	16836156	-1.216266	-2.32345	5.77E-05	0.0168235
SUV39H2	16702685	-1.016533	-2.02305	5.70E-05	0.0168235
ELAVL3	16868986	-0.966905	-1.95464	5.84E-05	0.0168235
NHLRC2	16709429	-0.879605	-1.83987	5.90E-05	0.0168235
MFN1	17125550	-0.960631	-1.94616	6.10E-05	0.0170189
EHD3	16878731	-0.905669	-1.87341	6.14E-05	0.0170189
CHAC1	16799739	0.912213	1.88193	6.23E-05	0.0170207
ACSL4	17113293	-0.822163	-1.76805	6.37E-05	0.0171726
TTC37	16998211	-1.120854	-2.17476	6.47E-05	0.0171908
CENPN	16821239	-1.007171	-2.00997	7.15E-05	0.0187545
MFN1	16948249	-0.928452	-1.90323	7.58E-05	0.0196176
C18orf54	16852445	-1.127683	-2.18508	8.33E-05	0.0199833
DCAF17	16887561	-1.088926	-2.12716	8.83E-05	0.0199833
SLC25A40	17059532	-1.057239	-2.08094	8.62E-05	0.0199833
FBXO8	16981588	-0.994925	-1.99298	8.96E-05	0.0199833
FAM111B	16725041	-0.94571	-1.92614	8.46E-05	0.0199833
ELK3	16755339	-0.927327	-1.90175	8.79E-05	0.0199833
RNF168	16963358	-0.858787	-1.81351	8.93E-05	0.0199833
DICER1	16796325	-0.855893	-1.80988	8.42E-05	0.0199833
SLC38A11	16904488	-0.82857	-1.77592	8.35E-05	0.0199833
SPAST	16878748	-1.00705	-2.0098	9.58E-05	0.0211239
ADAR	16693813	-1.079992	-2.11402	0.000107	0.0226068
LETMD1	16751209	-1.041352	-2.05816	0.000111	0.0226068
AAK1	16898617	-0.986923	-1.98195	0.000107	0.0226068
LINC01578	16805218	-0.964684	-1.95164	0.000109	0.0226068
SLC25A32	17079971	-0.904913	-1.87243	0.000105	0.0226068
PCLAF	16810559	-0.877928	-1.83773	0.000105	0.0226068
MMS22L	17021845	-1.015985	-2.02228	0.000115	0.0230761
FAM72A	16676526	-0.98256	-1.97597	0.000115	0.0230761

GEN1	16877473	-0.881238	-1.84196	0.000122	0.0238723
HACD2	16958236	-1.065999	-2.09362	0.000125	0.0241031
SLC39A8	16978417	-1.05701	-2.08061	0.000131	0.0246141
FAM72B	16669422	-0.94706	-1.92794	0.000136	0.0251218
PRKD3	16896502	-1.05452	-2.07703	0.000149	0.0256063
CAB39	16891971	-1.052752	-2.07448	0.000144	0.0256063
IRX6	16819052	-0.997705	-1.99682	0.000148	0.0256063
CENPW	17012379	-0.934878	-1.91173	0.000147	0.0256063
HIST1H1B	17016499	-0.879098	-1.83923	0.000144	0.0256063
CLSPN	16685165	-0.861898	-1.81743	0.000142	0.0256063
MIOS	17043550	-1.022092	-2.03086	0.000153	0.0261031
ITGB3BP	16688123	-1.052631	-2.07431	0.000162	0.0266104
RMI1	17086353	-0.965056	-1.95214	0.000158	0.0266104
FOXJ3	16685958	-0.891661	-1.85531	0.000159	0.0266104
CCNE1	16860418	-0.875596	-1.83477	0.000162	0.0266104
FASTKD1	16904917	-1.040641	-2.05714	0.000179	0.0281498
PHTF2	17047707	-1.015701	-2.02188	0.00018	0.0281498
DTL	16677201	-0.934787	-1.91161	0.000178	0.0281498
INTS8	17070998	-0.956666	-1.94082	0.000186	0.0283587
RASEF	17095194	-0.857423	-1.8118	0.000189	0.0283587
STC1	17075553	-0.864303	-1.82046	0.000193	0.0287895
UGDH	16975310	-0.95663	-1.94077	0.000201	0.029333
TMEM138	16725589	-0.924091	-1.89749	0.000205	0.0296709
SRP54	16783310	-1.085728	-2.12245	0.000211	0.0301332
SLC11A2	16764620	-1.06083	-2.08613	0.000229	0.0321858
ERI1	17065525	-1.045394	-2.06393	0.000234	0.0324308
TMED5	16689664	-0.962641	-1.94887	0.000239	0.0324308
TROVE2	16675334	-0.924014	-1.89739	0.000234	0.0324308
LMBRD2	16995254	-0.894165	-1.85853	0.000244	0.0324308
SEMA6A	16999083	-0.870595	-1.82842	0.000243	0.0324308
PAXIP1	17064724	-0.788415	-1.72718	0.00024	0.0324308
PMS2	17054923	-0.894594	-1.85909	0.000248	0.0324874
SLC7A6	16820398	-0.835429	-1.78439	0.000255	0.0329405
DEPDC1B	16996545	-0.790759	-1.72998	0.000259	0.0333082
JPT2	16814779	-0.951834	-1.93433	0.000264	0.0337525
MCM6	16903090	-0.958251	-1.94295	0.000285	0.0355312
PPFIBP1	16749459	-0.849533	-1.80192	0.00029	0.0358148
RFX5	16693057	-0.982293	-1.9756	0.000306	0.0375386
SKA3	16777278	-0.867793	-1.82487	0.000309	0.0377287
PPFIBP1	17120706	-0.846838	-1.79855	0.000315	0.0381846
CDC7	16667037	-0.962437	-1.9486	0.000319	0.0382511
DIAPH3	16779546	-0.846224	-1.79779	0.000327	0.0383709
ZBED6	16676263	-0.840702	-1.79092	0.000328	0.0383709
WASHC5	17080946	-0.828094	-1.77534	0.00033	0.0383709
SLC50A1	16671653	-0.84209	-1.79264	0.000342	0.0392104
AZI2	16951820	-1.073583	-2.10465	0.000363	0.040196
EXOSC2	17090357	-0.922435	-1.89531	0.00036	0.040196
MBD4	16958989	-0.883125	-1.84437	0.000362	0.040196
AKR1C2	16711343	-0.835606	-1.78461	0.000385	0.0408372
CENPH	16985614	-0.817129	-1.7619	0.000381	0.0408372

HELLS	16707695	-0.811285	-1.75477	0.000377	0.0408372
CENPK	16996722	-0.802539	-1.74417	0.000382	0.0408372
CEP55	16707551	-0.955344	-1.93904	0.000398	0.041752
SECISBP2	17086640	-0.916716	-1.88781	0.00042	0.0432112
KIF11	16707468	-0.883411	-1.84473	0.000421	0.0432112
TBC1D9	16980096	-0.798111	-1.73882	0.000417	0.0432112
SLC12A2	16988801	-0.890489	-1.8538	0.000433	0.0441729
PLAA	17092969	-0.916632	-1.8877	0.000441	0.0447991
GMNN	17005396	-0.881487	-1.84227	0.000463	0.0465645
SS18	16854375	-0.992179	-1.98919	0.000472	0.0467493
NUF2	16673154	-0.973241	-1.96325	0.000468	0.0467493
KIF23	16802519	-0.794515	-1.73449	0.000471	0.0467493
TOMM34	16919508	-0.844734	-1.79593	0.000489	0.0481901
CASP8AP2	17010991	-0.868583	-1.82587	0.000499	0.0486389
C12orf29	16754917	-0.84466	-1.79584	0.00051	0.0494716
PRDX3	16718922	-0.830776	-1.77864	0.000523	0.0499407
SEMA3C	17059119	-0.90331	-1.87035	0.000539	0.051215
ERGIC2	16762700	-0.884209	-1.84575	0.000541	0.051215
NAP1L1	16767756	-0.845367	-1.79672	0.000545	0.051319
TAB2	17013567	-0.843523	-1.79443	0.000563	0.0524442
EXO1	16679411	-0.842008	-1.79254	0.00056	0.0524442
FBXO5	17024980	-0.837046	-1.78639	0.000565	0.0524442
NR2C1	16768646	-0.944005	-1.92386	0.000574	0.0530686
EIF5A2	16961475	-0.89239	-1.85625	0.000585	0.0536902
HIST1H2BI	17005603	-0.801442	-1.74284	0.000587	0.0536902
CCNB1	16985599	-0.813724	-1.75774	0.000631	0.0564276
DBF4	17047965	-0.777625	-1.71431	0.000646	0.0575227
ARV1	16678680	-0.899764	-1.86576	0.000657	0.0577338
KNTC1	16758336	-0.85701	-1.81128	0.000661	0.0577338
CDC6	16834056	-0.770328	-1.70566	0.000658	0.0577338
HAUS4	16790592	-0.802098	-1.74363	0.000678	0.0589722
TMPO	16755498	-0.864499	-1.82071	0.000702	0.060164
ZGRF1	16979060	-0.77587	-1.71222	0.000704	0.060164
NDC1	16687418	-0.842377	-1.793	0.00072	0.060497
PDZD8	16718769	-0.820728	-1.7663	0.000723	0.060497
PIGA	17109260	-0.79143	-1.73079	0.000717	0.060497
GSTCD	16969473	-0.879484	-1.83972	0.000734	0.0607141
MGAT5	16885874	-0.823878	-1.77016	0.000739	0.0607141
FAM107B	16711952	-0.925196	-1.89894	0.000748	0.0607422
CNST	16679627	-0.801943	-1.74345	0.000783	0.0630586
GMCL1	16881098	-0.803827	-1.74573	0.000793	0.0635712
TERF1	17069989	-0.929016	-1.90398	0.000803	0.0637002
HMGN2	16661192	-0.846595	-1.79825	0.000808	0.0637002
OTUD4	16980311	-0.813801	-1.75784	0.000813	0.0638612
TMEM229A	17062479	-0.810353	-1.75364	0.000831	0.0645191
MND1	16971573	-0.816514	-1.76114	0.000841	0.0647484
DBF4	17120506	-0.794134	-1.73404	0.000849	0.0650963
FAM135A	17010104	-0.868709	-1.82603	0.000887	0.0665508
RAB39A	16730788	-0.816104	-1.76065	0.000888	0.0665508
HMMR	16991859	-0.788731	-1.72755	0.000887	0.0665508

USP12	16777602	-0.794205	-1.73412	0.000907	0.0675714
FAM120AOS	17095981	-0.857865	-1.81235	0.000933	0.0688312
R3HDM1	16885978	-0.77849	-1.71533	0.000962	0.0693939
ATP8A1	16975506	-0.873133	-1.83164	0.000969	0.0696182
FAM35A	16706933	-0.891686	-1.85534	0.001	0.0705308
SPPL2A	16809187	-0.925544	-1.8994	0.001033	0.0712989
NRDC	16687071	-0.893192	-1.85728	0.001036	0.0712989
FUBP1	16688753	-0.822158	-1.76805	0.001036	0.0712989
PHF10	17025937	-0.779648	-1.71671	0.001047	0.071553
SLC25A17	16935320	-0.865215	-1.82161	0.001072	0.0720607
WLS	16688339	-0.801267	-1.74263	0.001073	0.0720607
WDR76	16800355	-0.786046	-1.72434	0.001069	0.0720607
C3orf14	16942311	-0.917409	-1.88872	0.00113	0.0730311
SGO2	16889251	-0.837515	-1.78697	0.001127	0.0730311
SFR1	16708910	-0.777773	-1.71448	0.001146	0.0736525
DTX3L	16944665	-0.86284	-1.81861	0.00119	0.0738083
HIGD1A	16952639	-0.842878	-1.79362	0.001195	0.0738083
SGO1	16951485	-0.903819	-1.87101	0.00121	0.0743072
CHKA	16741201	-0.862703	-1.81844	0.00121	0.0743072
PIAS3	16669905	-0.787811	-1.72645	0.001236	0.0753913
KAT6A	17076694	-0.773505	-1.70942	0.001334	0.0794155
PBK	17075776	-0.88647	-1.84865	0.001341	0.0795702
HIF1A	16785058	-0.88782	-1.85038	0.001396	0.0798295
RPA3	17055191	-0.878008	-1.83784	0.001403	0.0798295
CTDSPL2	16800406	-0.835611	-1.78461	0.001365	0.0798295
SNX5	16917504	-0.82603	-1.7728	0.001394	0.0798295
LRRC49	16802605	-0.787801	-1.72644	0.001394	0.0798295
ZBTB38	16946366	-0.780825	-1.71811	0.001403	0.0798295
UNC5C	16978054	-0.898	-1.86348	0.001437	0.0809666
DNAH14	16677977	-0.931106	-1.90674	0.001474	0.0813954
PPEF1	17101923	-0.864962	-1.82129	0.001467	0.0813954
DRAM2	16690769	-0.836893	-1.7862	0.001484	0.081575
KBTBD2	17056493	-0.907755	-1.87612	0.0015	0.081587
NUP37	16769227	-0.903044	-1.87001	0.001508	0.081587
PSMG1	16925674	-0.884291	-1.84586	0.001507	0.081587
CEP44	16972480	-0.833363	-1.78183	0.001522	0.081587
MLH1	16938899	-0.766452	-1.70108	0.001518	0.081587
TATDN1	17080869	-0.843619	-1.79455	0.001543	0.0819412
TYW3	16666204	-0.811725	-1.75531	0.001537	0.0819412
BTG3	16924305	-0.854158	-1.8077	0.001649	0.0851165
SMG8	16836504	-0.852885	-1.80611	0.001661	0.0851165
DDHD2	17068014	-0.867893	-1.825	0.001835	0.089904
PARPBP	16755928	-0.833325	-1.78179	0.001833	0.089904
GABRB1	16966497	-0.835684	-1.7847	0.001947	0.0926235
CHRNA5	16803562	-0.790759	-1.72998	0.001978	0.093177
GTF2H3	16758650	-0.835612	-1.78461	0.001995	0.0937412
CDKL5	17101896	-0.77207	-1.70772	0.002003	0.0938523
LRRC37A	16835045	-0.792353	-1.7319	0.00201	0.093952
INTS7	16699021	-0.847616	-1.79952	0.002093	0.0953093
ECT2	16948021	-0.826983	-1.77397	0.002092	0.0953093

PTPRO	16748711	-0.770099	-1.70539	0.002103	0.0953218
STXBP4	16836232	-0.805737	-1.74804	0.002141	0.0968209
DNM1L	16749826	-0.873532	-1.83214	0.002191	0.0980188
TAF1B	16876950	-0.786747	-1.72518	0.00221	0.0983411
RALA	17045369	-0.854622	-1.80829	0.002307	0.0996013
KIAA0368	17097152	-0.81619	-1.76075	0.002415	0.0998341
ICE2	16810104	-0.806559	-1.74903	0.002386	0.0998341
SCG2	16908977	-0.802284	-1.74386	0.002393	0.0998341
ACOT13	17005385	-0.80227	-1.74384	0.002368	0.0998341
PLS3	17106357	-0.779951	-1.71707	0.002444	0.0998341
ARFGEF2	16914628	-0.767929	-1.70282	0.002411	0.0998341
NT5C3A	17056515	-0.875369	-1.83448	0.002486	0.1009027
PSMG1	16925668	-0.883238	-1.84451	0.002546	0.1021945
DMXL1	16988301	-0.788288	-1.72702	0.002759	0.1069347
UTP15	16986117	-0.836415	-1.78561	0.002805	0.1079846
ARHGAP42	16730463	-0.787897	-1.72656	0.00311	0.1127497
ZMYM1	16662338	-0.766539	-1.70118	0.003224	0.1145525
ZBTB24	17022465	-0.773383	-1.70927	0.003285	0.1152386
ACTR3	16884797	-0.789043	-1.72793	0.003758	0.1227648
NXF1	16739552	-0.822494	-1.76846	0.004203	0.1296744
TRO	17104051	-0.783464	-1.72126	0.004327	0.1315773
SMC2	17087716	-0.768011	-1.70292	0.00544	0.1460707
CLOCK	16976074	-0.788196	-1.72691	0.00562	0.1471072

Supplementary Table 3. List of transcription factor target motifs around transcription start sites of genes down-regulated by lncNB1 siRNAs. Genome-wide differential gene expression studies were performed with Affymetrix microarray in BE-(2) cells 40 hours after transfection with control siRNA, lncNB1 siRNA-1 or lncNB1 siRNA-2. Gene set enrichment analysis generated enriched gene sets of transcription factor target motifs around transcription start sites of genes down-regulated by lncNB1 siRNAs. Size represented the number of genes down-regulated by lncNB1 siRNAs in each gene set, ES and NES enrichment score and normalized enrichment score. Gene sets with normalized p values and false discovery rates (FDR) < 0.01 were listed.

NAME	NOM p-value	ES	NES	SIZE
E2F_Q4_01	0.001092896	-0.62267	-2.35612	202
E2F1_Q4_01	0.001091703	-0.62262	-2.35483	201
E2F1_Q6_01	0.001101322	-0.62086	-2.35204	210
E2F_Q6	0.001092896	-0.62133	-2.34794	200
E2F_Q3_01	0.001097695	-0.61966	-2.34732	205
E2F_Q3	0.001097695	-0.618	-2.33303	196
E2F1_Q6	0.001092896	-0.61602	-2.32785	200
E2F_Q4	0.001092896	-0.61244	-2.31432	200
E2F1DP1RB_01	0.001113586	-0.60758	-2.28575	194
E2F4DP1_01	0.001092896	-0.60016	-2.27094	202
E2F1_Q3	0.001097695	-0.59277	-2.24547	205
E2F_Q6_01	0.001096491	-0.59166	-2.24287	208
E2F1DP1_01	0.00109529	-0.5917	-2.23624	198
E2F1DP2_01	0.00109529	-0.5917	-2.23624	198
E2F4DP2_01	0.00109529	-0.5917	-2.23624	198
E2F_03	0.001104972	-0.58867	-2.2299	211
E2F_02	0.00109529	-0.58924	-2.22694	198
SGCGSSAAA_E2F1DP2_01	0.001150748	-0.59989	-2.20498	141
E2F_01	0.001295337	-0.6485	-2.14728	61
GCCATNTTG_YY1_Q6	0.001029866	-0.54513	-2.14333	374
E2F1_Q4	0.001101322	-0.56167	-2.13357	217
NFMUE1_Q6	0.00109529	-0.54817	-2.08024	207
YY1_Q6	0.001097695	-0.49655	-1.88164	206
AHR_Q5	0.001112347	-0.49952	-1.87914	188
GABP_B	0.001094092	-0.4691	-1.78795	230
YY1_02	0.001101322	-0.46935	-1.77808	210
ELK1_02	0.001101322	-0.46043	-1.74899	217
E2F1_Q3_01	0.001102536	-0.4605	-1.74817	220
USF_C	0.001078749	-0.45444	-1.74067	248
NMYC_01	0.001089325	-0.45182	-1.72653	236
NRF1_Q6	0.001103753	-0.45283	-1.71689	213
ARNT_01	0.001102536	-0.44633	-1.69538	219
NFY_C	0.001102536	-0.44413	-1.68626	218
NFY_Q6_01	0.001091703	-0.42673	-1.63045	237
USF2_Q6	0.001103753	-0.42655	-1.61897	222
USF_01	0.001098901	-0.42326	-1.6092	226
MAX_01	0.001102536	-0.42401	-1.60879	223

ARNT_02	0.001103753	-0.42123	-1.59706	213
SP1_Q4_01	0.001098901	-0.41769	-1.58803	226
HIF1_Q3	0.00109529	-0.41649	-1.57403	198
MYCMAX_03	0.001102536	-0.41438	-1.574	219
YY1_01	0.001102536	-0.41422	-1.57245	221
CMYB_01	0.001101322	-0.41329	-1.5657	210
MAF_Q6	0.001094092	-0.40271	-1.53493	230
MYCMAX_01	0.001101322	-0.40218	-1.52712	225
NRF2_01	0.001085776	-0.39709	-1.51823	234
MYCMAX_02	0.001096491	-0.38736	-1.47387	227
NFY_Q6	0.001086957	-0.38425	-1.46838	233

Supplementary Table 4. lncNB1 RNA-binding proteins identified by RNA-binding protein pull-down assays and mass spectrometry analysis.

Protein ID	Protein name	Molecular weight (KDa)	Score *	Matches †	Sequences †	*emPAI* ‡
gi|45446747	ATP-dependent RNA helicase DDX42	102.912	96	4 (4)	4 (4)	0.14
gi|5174449	Histone H1X	22.474	52	4 (4)	4 (4)	0.83
gi| 48145659	Heterogeneous nuclear ribonucleoprotein K	50.944	173	8 (8)	7 (7)	0.61
gi| 392513662	Interleukin enhancer-binding factor 2	43.035	114	5 (5)	5 (5)	0.49
gi| 48145871	60S ribosomal protein L35	14.543	56	3 (3)	3 (3)	0.99

*Score: total score from each identified peptide per protein

†Matches/Sequences: numbers outside parenthesis indicates the number of identified peptides/sequences per protein, number in parenthesis indicates the number of peptides/sequences identified that match the corresponding protein.

‡ emPAI (exponentially modified protein abundance index): 10 to the power of PAI minus 1 (10PAI–1). PAI is determined by dividing the number of experimentally identified peptides by the total number of possible observable peptides per protein.

Supplementary Table 5. mRNAs down-regulated in the heavy polysome after lncNB1 knockdown. BE(2)-C cells were transfected with control siRNA, lncNB1 siRNA-1 or lncNB1 siRNA-2, followed by treatment with 50 µg/ml of cycloheximide and polysome fractionation. RNA was extracted from the heavy polysome and subjected to Affymetrix microarray analysis. The experiments were repeated three times. Differential expression analysis was performed using the Limma package. Moderated t tests were performed with the Limma package. mRNAs with fold change of 1.5 and $p < 0.05$ were listed.

Probe ID	Gene Symbol	Fold Change	logFC	Average Expression	t t	P Value	Adjusted P Value
TC0900009948.hg.1	ARHGEF39	-1.52026	-0.6043	7.7118313	-4.4382	0.0007	0.243907
TC0Y00007236.hg.1	CDY1B	-1.49466	-0.5798	4.4989103	-4.49262	0.0007	0.243907
TC1700010973.hg.1	COPZ2	-1.48438	-0.5699	5.3666265	-4.81177	0.0004	0.195389
TC0300011276.hg.1	DENND6A	-2.02427	-1.0174	8.4120972	-6.65154	2E-05	0.051845
TC1000007641.hg.1	DKK1	-2.40744	-1.2675	6.9090121	-8.53193	1E-06	0.010177
TC0200015894.hg.1	DOCK10	-1.7258	-0.7873	4.1767419	-4.8209	0.0004	0.195389
TC2000008894.hg.1	E2F1	-1.46799	-0.5538	8.3847022	-4.28421	0.001	0.256876
TC0800009600.hg.1	FAM167A	-1.77617	-0.8288	8.9222414	-4.49228	0.0007	0.243907
TC1700012183.hg.1	FGF11	-1.60074	-0.6787	7.0117357	-6.19995	4E-05	0.075862
TC0100013882.hg.1	FOXJ3	-1.4871	-0.5725	9.8273065	-4.36342	0.0008	0.254443
TC0500009131.hg.1	G3BP1	-1.93305	-0.9509	9.4198687	-6.00384	5E-05	0.086867
TC0100007512.hg.1	GPR3	-1.46573	-0.5516	6.9616362	-5.17362	0.0002	0.135984
TC0100009480.hg.1	HIPK1	-1.75387	-0.8105	10.037117	-5.01462	0.0003	0.156341
TC0400011548.hg.1	LEF1	-1.46432	-0.5502	7.7329734	-5.6145	1E-04	0.099777
TC1200008176.hg.1	LGR5	-1.87792	-0.9091	6.1321785	-4.477	0.0007	0.243907
TC1000007092.hg.1	MASTL	-1.51021	-0.5947	8.2622669	-5.12183	0.0002	0.135984
TC0600012631.hg.1	MMS22L	-1.47126	-0.5571	9.0198649	-4.27596	0.001	0.256876
TC0600013271.hg.1	MTFR2	-1.52126	-0.6053	6.0928783	-4.26915	0.001	0.256876
TC1200010946.hg.1	NEMP1	-1.4554	-0.5414	9.7206464	-5.76828	8E-05	0.086867
TC1500009148.hg.1	OIP5	-1.54992	-0.6322	7.5747844	-4.78143	0.0004	0.199982
TC1600007718.hg.1	ORC6	-1.55982	-0.6414	8.4351528	-4.54829	0.0006	0.240301
TC1100012094.hg.1	PGR	-1.86263	-0.8973	4.9751901	-4.25593	0.001	0.256876
TC0X00009124.hg.1	PIGA	-1.51518	-0.5995	8.3873017	-6.60872	2E-05	0.051845
TC1500009756.hg.1	RBPMS2	-1.72367	-0.7855	8.5257932	-5.13437	0.0002	0.135984
TC0300013703.hg.1	RNF168	-1.70829	-0.7726	8.6991174	-4.74811	0.0004	0.204536
TC0800008299.hg.1	SDC2	-1.47839	-0.564	10.671754	-5.2446	0.0002	0.130173
TC0700011710.hg.1	SLC25A40	-1.67846	-0.7471	7.0943175	-5.91314	6E-05	0.086867
TC2000008023.hg.1	SLCO4A1	-1.64195	-0.7154	7.5253387	-6.47034	3E-05	0.058044
TC1500010880.hg.1	TIPIN	-1.75079	-0.808	8.6749407	-5.1886	0.0002	0.135984
TC1500010946.hg.1	TM2D3	-1.74411	-0.8025	6.2743989	-4.7098	0.0005	0.214967
TC1100007801.hg.1	TMEM138	-1.73006	-0.7908	9.8561454	-4.93524	0.0003	0.171891
TC1400008591.hg.1	TMEM55B	-1.52455	-0.6084	6.915402	-4.59578	0.0006	0.237244
TC1400009184.hg.1	TXNDC16	-1.50469	-0.5895	7.9926854	-4.29112	0.001	0.256876
TC0300009613.hg.1	USP13	-1.7387	-0.798	7.5634111	-4.27841	0.001	0.256876

Supplementary Table 6. Sequences of oligoes.

**Oligonucleotides**	SOURCE	IDENTIFIER
PCR primers for RNA immunoprecipitation-PCR		
lncNB1-forward (F): 5'-AATACGCCAATGTCCTGCTC-3'<br>lncNB1-reverse (R): 5'-TTTCCAGTGTCCTTCGAACC-3'	This paper	N/A
Primers for amplifying full length lncNB1 cDNA containing T7 and SP6 promoters		
lncNB1T7-F:<br>taatacgactcactatagggagaTTCCTGTCATGTGAAACATG<br>lncNB1SP6-R:<br>atttaggtgacactatagaagggGGCCAACAACTGTTTAATG	This paper	N/A
Real time RT-PCR primers		
Hs_E2F1-F: 5'-GGACTCTTCGGAGAACTTTCAGAT-3'<br>Hs_E2F1-R: 5'-GGGCACAGGAAAACATCGAT-3'	This paper	N/A
Hs_N-Myc-F: 5'-CGACCACAAGGCCCTCAGTA-3'<br>Hs_N-Myc-R: 5'-CAGCCTTGGTGTTGGAGGAG-3'	This paper	N/A
Hs_lncNB1-F: 5'-AATACGCCAATGTCCTGCTC-3'<br>Hs_lncNB1-R: 5'-TCAGTGCCTTGGCTTGTAGA-3'	This paper	N/A
Hs_DEPDC1B-F: 5'-AGCCTTGTTGGAGGAAGTCA-3'<br>Hs_DEPDC1B-R: 5'-TTTCGGTTTGGGTTTTTCAG-3'	This paper	N/A
Hs_TUBB2A-F: 5'-AATGAGGCTGCTGGTAACAAA-3'<br>Hs_TUBB2A-R: 5'-AAGGGTCCAGACCTGACAGA-3'	This paper	N/A
Hs_TUBB2B-F: 5'-AGGACGGACAGACCCAGAC-3'<br>Hs_TUBB2B-R: 5'-CAATCCCATGCTCATCACTG-3'	This paper	N/A
Hs_ILF2-F: 5'-TCCGAATCCTCTCACATGGT-3'<br>Hs_ILF2-R: 5'-CCTTCTTCTCTGGTGGCTTC-3'	This paper	N/A
Hs_HNRPK-F: 5'-GACGGCATGGTTGGTTTCA-3'<br>Hs_HNRPK-R: 5'-ATTCTGATGGGCTCCATGTATCTAT-3'	This paper	N/A
Hs_H1X-F: 5'-TGGGCGCACCTACCTCAA-3'<br>Hs_H1X-R: 5'-CCTGCAGAAGCGTGTCGTT-3'	This paper	N/A
Hs_DDX42-F: TGAGAACATGGATCGAGGAAATAA<br>Hs_DDX42-R: TCTCCCATAGCTCCTGTGGAA	This paper	N/A
Hs_E2F1-F: 5'- GGACTCTTCGGAGAACTTTCAGAT-3'<br>Hs_E2F1-R: 5'- GGGCACAGGAAAACATCGAT-3'	This paper	N/A
Hs_RPL35-F: 5'-CGGTGCGGCCTCCAA-3'<br>Hs_RPL35-R: 5'-CACGGGCAATGGATTTCC-3'	This paper	N/A
Hs Actin-F: 5'-AGGCCAACCGCGAGAAG-3'<br>Hs Actin-R: 5'-ACAGCCTGGATAGCAACGTACA-3'	This paper	N/A
Hs_MALAT1-F: 5'-GACGGAGGTTGAGATGAAGC-3'<br>Hs_MALAT1-R: 5'-ATTCGGGGCTCTGTAGTCC-3'	This paper	N/A
Hs_GAPDH-F: 5'-CATGGGGAAGGTGAAGGTC-3'<br>Hs_GAPDH-R: 5'-AACAATATCCACTTTACCAGAGTT-3'	This paper	N/A
Promoter primers for ChIP PCR		
DEPDC1B negative control-F:<br>5'-AGAAGTCTGGGAAGGGTGCT-3'<br>DEPDC1B negative control-R:<br>5'-ATGCCAGCTTCTTGAGCATT-3'	This paper	N/A

DEPDC1B gene promoter-F: 5’-GTTTCGGTCGCTGGATAACA-3’<br>DEPDC1B gene promoter-R: 5’-CTAGGCAGGTGCGACTAAGG-3’	This paper	N/A
E2F1 negative control-F: 5’-GCAGATGAGGCAAGCAAAGC-3’<br>E2F1 negative control-R: 5’-CCATCCAAAAGGCAGTCTAACAT-3’	This paper	N/A
E2F1 promoter-F: 5’-AATGTCATGGGTGAGGCAAGTT-3’<br>E2F1 promoter-R: 5’-CAACCTGTAGCCCCCAACAG-3’	This paper	N/A
DEPDC1B TSS-F: 5’-CTTGGGATTCAAACTGCTGCTC-3’<br>DEPDC1B TSS-R: 5’-CACGATGCGATGCTCCATGG-3’	This paper	N/A
DEPDC1B-600bp-F: 5’-CCCTCTGAAAGATTGCAAATCGG-3’<br>DEPDC1B-600bp-R: 5’-TTAGTCCTCGGCAATAGGGTTG-3’	This paper	N/A
DEPDC1B+600bp-F: 5’-GACGGGGTATTCGAATAAACGG-3’<br>DEPDC1B+600bp-R: 5’- GGTGCTGACAGAAACAGAAACG -3’	This paper	N/A
DEPDC1B-3500bp control-F:<br>5’-CATGCTGGGCACTATATTTAGCAC-3’<br>DEPDC1B-3500bp control-R:<br>5’-CCATCTACTCACTGGTAAACTGG -3’	This paper	N/A
Primers for generating wild type and 20bp deletion DEPDC1B gene promoter pGL3 constructs		
1146bp promoter-F:<br>5’-CTCGAGTTAACTTCCACAGCTCACAAAG-3’<br>1146bp promoter-R:<br>5’-GAGCAGCAGTTTGAATCCCAAG-3’	This paper	N/A
545bp promoter-F: 5’-TCGGGGCTCCCTTCCCGC-3’<br>545bp promoter-R: 5’-GGTACCTATCGATAGAGAAATG-3’	This paper	N/A
75bp promoter-F: 5’-ATTTCCGGTGACGTGCTG-3’<br>75bp promoter-R: 5’-GGTACCTATCGATAGAGAAATG-3’	This paper	N/A
545bpPromoter+20bpDeletion-F:<br>5’-GGGATTCAAACTGCTGCTCAGATCTGC-3’<br>545bpPromoter+20bpDeletion-R:<br>5’-TGATTGGGCGGCGCGGCA-3’	This paper	N/A
75bpPromoter+20bpDeletion-F:<br>5’-TGGGATTCAAACTGCTGCTCAGATCTGC-3’<br>75bpPromoter+20bpDeletion-R: 5’-TGATTGGGCGGCGCGGCA-3’	This paper	N/A
Primers used obtain E2F1 and DP1 coding sequences for cloning into mammalian expression vectors		
pCMV14-HindIII E2F1-F:<br>5’-TTTAAGCTTatggccttggccggggcccctg-3’	This paper	N/A
pCMV14-ECORI E2F1-R:<br>5’-TTTGAATTCtcagaaatccagggggggtgaggtccccaaag-3’	This paper	N/A
pCMV10-HindIII DP1-F:<br>5’-TTTAAGCTTgcaaaagatgccggtctaattgaag-3’	This paper	N/A
pCMV10-XbaI DP1-R:<br>5’- TTTTCTAGAgtcgtcctcgtcattctcgttg-3’	This paper	N/A
siRNA target sequences		
All star negative control	Qiagen	Cat#1027281
RPL35 siRNA-1: 5’-CCGTGTTCTCACAGTTATTAA-3’	Qiagen	Cat#SI02663871
RPL35 siRNA-2: 5’-TGCAGCAATGGCCAAGATCAA-3’	Qiagen	Cat#SI02663878
E2F1 siRNA-1: 5’-CTCACTGAATCTGACCACCAA-3’	Qiagen	Cat#SI00073997

E2F1 siRNA-2: 5’-CAGATCTCCCTTAAGAGCAAA-3’	Qiagen	Cat#SI03064775
DDX42 siRNA-1: 5’-CCCGGCGTCTGGTAGAATTTA-3’	Qiagen	Cat#SI04156936
DDX42 siRNA-2: 5’-TACTAAGGTGTCACCTTATAA-3’	Qiagen	Cat#SI05037151
DEPDC1B siRNA-1: 5’-GAGGAGCGTGTGGCTCATCTA-3’	Qiagen	Cat#SI04341638
DEPDC1B siRNA-2: 5’-GAGTTATTAGCTGCTAGATTGGTAA-3’	Invitrogen	Cat#272497B09
H1X siRNA-1: 5’-CGCCGAGGGAATGGCCAAGAA-3’	Qiagen	Cat#SI00432985
H1X siRNA-2: 5’-CTCGTCGCTGGCCAAGATCTA-3’	Qiagen	Cat#SI04156810
ILF2 siRNA-1: 5’-CAGGCCCTTTGTACCACATAT-3’	Qiagen	Cat#SI02664844
ILF2 siRNA-2: 5’-CTCCATAGAAGTCATTCCA-3’	Qiagen	Cat#SI02664851
HNRPK siRNA-1: 5’-TGGCATTTGTATTGATAGTTA-3’	Qiagen	Cat#SI05034162
HNRPK siRNA-2: 5’-TATGTTAGTTGTGAAGAACTA-3’	Qiagen	Cat#SI04216618
lncNB1 siRNA-1: 5’-CAGCTGCAGCGTTTACCCAAA-3’	Qiagen	Cat#SI05715486
lncNB1 siRNA-2: 5’-CACAGCGAATGCTAACTGATA-3’	Qiagen	Cat#SI05715500
shRNA target sequences		
Control shRNA: 5’-GCACTACCAGAGCTAACTCAGATAGTACT-3’	This paper	N/A
lncNB1 shRNA1: 5’- GCTTCCTTCAAACCTCAAATC-3’	This paper	N/A
lncNB1 shRNA-2: 5’-GCTGCAGCGTTTACCCAAAGA-3’	This paper	N/A
