# Supplementary material for: The long noncoding RNA lncNB1 promotes tumorigenesis by interacting with ribosomal protein RPL35
Source: Nat Commun. 2019 Nov 5;10:5026. doi: 10.1038/s41467-019-12971-3 (PMC6831662; doi:10.1038/s41467-019-12971-3)
Supplement: Supplementary file 3 — Description of Additional Supplementary Files [file 41467_2019_12971_MOESM3_ESM.pdf]

Description of Additional Supplementary Files

File Name: Supplementary Data 1
Description: Genes differentially expressed between four MYCN gene-amplified [BE(2)-C, Kelly, CHP134 and SK-N-DZ] and two MYCN-non-amplified (SY5Y and SK-N-AS) neuroblastoma cell lines, as identified by RNA sequencing. FC and CPM represented fold change and counts per million reads, and FDR represented false discovery rate. Transcripts with an absolute log2 fold change of >1 and a false discovery rate of <0.05 were listed.
